# Bias and Modality in Conditionals: Experimental Evidence and Theoretical Implications

**DOI:** 10.1007/s10936-021-09813-z

**Published:** 2021-11-01

**Authors:** Mingya Liu, Stephanie Rotter, Anastasia Giannakidou

**Affiliations:** 1 Department of English and American Studies, Humboldt University of Berlin, Unter den Linden 6, 10099 Berlin, Germany; 2 Department of Linguistics, University of Chicago, Rosenwald 201A, Chicago, USA

**Keywords:** Bias, Modal verb, Adverb, Conditional connective, Questions, Conditionals, Experiment, German

## Abstract

The concept of bias is familiar to linguists primarily from the literature on questions. Following the work of Giannakidou and Mari (Truth and Veridicality in Grammar and Thought: Modality, Mood, and Propositional Attitudes, University of Chicago Press, Chicago, 2021), we assume “nonveridical equilibrium” (implying that *p* and *¬p* as equal possibilities) to be the default for epistemic modals, questions and conditionals. The equilibrium of conditionals, as that of questions, can be manipulated to produce bias (i.e., reduced or higher *speaker commitment*). In this paper, we focus on three kinds of modal elements in German that create bias in conditionals and questions: the adverb *wirklich* ‘really’, the modal verb *sollte* ‘should’, and conditional connectives such as *falls* ‘if/in case’. We conducted two experiments collecting participants’ inference about speaker commitment in different manipulations, Experiment 1 on *sollte/wirklich* in *ob-*questions and *wenn-*conditionals, and Experiment 2 on *sollte/wirklich* in *wenn/falls/*V1-conditionals. Our findings are that both *ob-*questions and *falls-*conditionals express reduced speaker commitment about the modified (antecedent) proposition in comparison to *wenn-*conditionals, which did not differ from V1*-*conditionals. In addition, *sollte/wirklich* in the antecedent of conditionals both create negative bias about the antecedent proposition. Our studies are among the first that deal with bias in conditionals (in comparison to questions) and contribute to furthering our understanding of bias.

## Introduction: Equilibrium and Bias in Questions and Conditionals

The concept of bias is familiar to linguists primarily from the literature on questions. While a plain question such as (1) simply seeks information, the questions in (2) are famously said to exhibit positive or negative bias:

**Table:** 

(1)	Is Agnes a vegetarian?	
(2)	a. Isn’t Agnes a vegetarian?	(high negation: positive bias)
	b. Agnes is vegetarian, isn’t she?	(negative tag: positive bias)
	c. Is Agnes really a vegetarian?	(adverb *really*: negative bias)

A speaker uttering (1) is in a state of *ignorance*: they don’t know if Agnes is vegetarian and ask (1) in order to find out. The polar question is therefore ‘information seeking’. This neutral state of ignorance is nonveridical, and has been characterized as being in *nonveridical equilibrium*:

**Table:** 

(3)	**Nonveridical equilibrium** (= ‘True uncertainty’ in Giannakidou, 2013)	
	A partitioned (*p* and *¬p*) epistemic or doxastic space M(i) is in nonveridical equilibrium if there is no bias; i is the individual anchor, by default the speaker.	

Nonveridical equilibrium says that *p* and *¬p* (i.e., not *p*) are equal possibilities, none is privileged over the other (Giannakidou, 2013; Giannakidou & Mari, 2018a, b, 2021a, b). The speaker has no preference for a positive or negative answer, no prior beliefs, knowledge, or expectations that would make them think that Agnes is or is not a vegetarian. Following the literature, we take equilibrium to be the default for epistemic possibility, questions, and conditionals.

What is called ‘bias’ is the destruction of equilibrium in a positive or negative direction. If the speaker adds certain devices, such as high negation (2a), a negative tag (2b), or the adverb *really* (2c), the questions are now said to favor a particular (positive or negative) answer (Sadock, 1971; Ladd, 1981; Abels, 2003; van Rooy & Šafárová 2003; Romero & Han, 2004; Reese, 2006; Reese & Asher, 2006; Krifka, 2015; Malamud & Stephenson, 2015; Farkas & Roelofsen, 2017; AnderBois, 2019; Mari & Tahar, 2019; Giannakidou & Mari, 2021a, b; Bill & Koev, 2021). In (2a,b) the speaker has a *positive* bias: the speaker seems to believe that Agnes is a vegetarian, and asks the question with the intention for their belief to be confirmed by the hearer. Likewise, in (2c), by adding *really,* the speaker intends to show that they don’t believe that Agnes is a vegetarian, and in this case, we talk about *negative* bias. Bias thus relies on assumptions the speaker makes prior to asking. Bias is also observed if the speaker chooses to add a negative polarity item (NPI) such as *even* in (4a) or in (4b) a minimizer NPI *lift a finger* (see Borkin, 1971; Giannakidou, 1997, 2007; van Rooy, 2003; Guerzoni, 2004; Guerzoni & Sharvit, 2007, a.o.).

**Table:** 

(4)	a. Have you spoken to Mary even once?	(NPI: negative bias)
	b. Did Mary lift a finger to help?	(NPI: negative bias)

These questions are negatively biased: the speaker disbelieves that the addressee has spoken to Mary or that Mary helped. In both cases, bias arises arguably because the speaker decides to use not a simple unadorned question, but to augment the basic option with the use of particular devices (or even mere intonation such as with rising declarative questions, falling interrogatives, etc.).

Crucially, we talk about *speaker bias* rather than question bias because bias is rooted in the speaker’s choice to go beyond the equilibrium information-seeking mode.^1^ Bias reflects the intrusion of the speaker’s assumptions and expectations (i.e., for the audience to agree with them), and is, of course, optional: the speaker doesn’t have to choose the biased option, see (1) vs. (2), or (4) vs. the sentences without NPIs. They do so because they have reasons to be more committed to the positive proposition (positive bias) or the negative one (negative bias). Bias, then, encodes the speaker’s doxastic and epistemic commitments that force them to abandon neutrality (presupposing, of course, that they are sincere). In the case of positive bias, the speaker seems to be *more committed* to *p*, and in the negative bias *less committed* to *p*. Speaker commitment^2^ is a notion we borrow from the modality vocabulary of Giannakidou and Mari (2018a, b, 2021a, b)’s veridicality theory that we discuss in Sect. 2, and is a handy way to talk about the speaker’s attitude towards the veridicality of a sentence.

The same manipulation of nonveridical equilibrium by speaker commitment is observed with conditionals, and it involves the use of modal devices such as tense and mood choices (see a.o., Iatridou, 1991; Fintel, 1999, 2007, 2011; Arregui, 2005), evaluative adverbs and discourse particles (Grosz, 2012; Liu, 2012) and NPIs (Liu, 2019, 2021). A hitherto less studied area concerns the choice of conditional connectives (CCs), see e.g., Ippolito & Su (2014) and Jiang (2019) on the Mandarin counterfactual CC *yaobushi* ‘if-not’, Hoeksema (2012) on *unless*, and Reis & Wöllstein (2010) and Liu (2019, 2021) on the German CCs *wenn* vs. *falls.*

**Table:** 

(5)	*Wenn/Falls*	*es*	*draußen*	*regnet,*	*bleibt*	*Susanne*	*zu*	*Hause.*	(CC *falls*: negative bias)
	if	it	outside	rains	stays	Susanne	at	home	
	‘If it is raining outside, Susanne will stay at home.’								

According to Liu (2019), the at-issue content of (5) is that of the regular conditional in both cases, but *falls* adds to the sentence a non-at issue content that the speaker takes it to be unlikely or does not take it to be likely that it is raining outside.^3^ Liu continues that *falls* expresses a weaker speaker commitment towards the antecedent than *wenn* and indicates that the speaker does not take *p* as *likely*. Liu (2021) discusses a number of diagnostics to make negative bias clear, of which we will mention one: *wenn* but not *falls* can be used in factual (premise) conditionals, as shown in (6).^4^

**Table:** 

(6)	A:	*Kai ist krank*.	(‘Kai is ill.’)					
	B:	*Wenn/#Falls*	*Kai krank *	*ist,*	*muss*	*das *	*Seminar*	*ausfallen*
		if	Kai ill	is	must	the	seminar	be cancelled
		‘Wenn/#Falls Kai is ill, the seminar must be cancelled.’						

*Falls* cannot be used if it is known that the antecedent is true or if the speaker intends to accommodate the antecedent proposition; rather, the choice of using *falls* instead of *wenn* conveys a negative bias. The *falls* vs. *wenn* contrast is observed in other languages too (such as Italian or Chinese, which we elaborate on later), and is correlated with the fact that many CCs have a modal source, e.g., the Modern Greek *an/ean* ‘if’ (Chatzopoulou, 2019, 2021), which starts out as a modal particle in Homeric and Classical Greek.

In conditionals, modal verbs and modal adverbs can also be used. As shown in the naturally-occurring examples across three different languages (English, German and Chinese) in (7), the presence of modal verbs *should/sollte/yao* and the adverb *really/wirklich/zhende* seems to convey a lower-than-equilibrium degree of speaker commitment towards the antecedent proposition, in comparison to their unmodalized variants such as *If it comes to this, then farewell, humanity!* in (7a).

**Table:** 

(7)	Modal verbs / adverb *really*: negative bias												
	a.	If it should really come to this, then farewell, humanity!^5^											
	b.	*Wenn*	*er*	*wirklich*	*im*	*Lotto*	*gewinnen*	*sollte,*	*fress*	*ich*	*einen*	*Besen.* 6	
		if	he	really	in the	Lottery	win	should	eat	I	a	broomstick	
		‘If he really wins the lottery, I'll eat a broomstick.’											
	c.	*Ruguo*	*ta*	*yao*	*zhende*	*xihuan*	*ni,*	*…*	*ta*	*jiu*	*yinggai*	*kefu*	*yixia.* 7
		if	he	should	really	like	you	…	he	JIU	should	overcome	once
		‘If he should really like you,…he should try to overcome it.’											

In this paper, we will focus on the interpretive effects of modal verbs, the adverb *really* and CCs in conditionals. For German, Reis & Wöllstein (2010: 137) have also noted the following relation between the modal verb *sollte* and the CC *falls*: “But what we can already say now is that *sollte* does roughly the same thing as *falls*: *falls* is also strictly limited to hypothetical conditionals and excludes counterfactual use^8^; *falls-*conditionals are often also marked with *sollte* (Zifonun et al., 1997: 2281). In particular, *falls,* via its literal meaning ‘in the case that …’, refers to the possibility of the realization of the antecedent proposition, just as *sollte* does.”^9^ The CC then is also understood, as we are suggesting, in modal terms.

While the intuition is clear that with modal modifications the equilibrium is manipulated by the speaker to reveal that they consider *p* less likely (or they are less certain about *p,* which we will get back to when we discuss the nature of the bias), these facts have hardly been tested experimentally in order to gain clarity about the size of the effects and interactions. We set out to examine the effect of modal elements in creating bias in this paper. Is the effect observable? How does negative bias arise? What is the contribution of the modal verbs and what is the contribution of the adverbs? How do they interact with each other and with conditionals?

The present paper is an attempt to address these questions and to draw some preliminary conclusions that can guide further research. The modality strategies observed in German—which we focus on from now on— make it necessary to assume that there is a modal structure in the conditional, following Giannakidou (2021) (see Kaufmann, 2005 for an earlier analysis to that end), and that this modal structure is responsible for the bias. In Sect. 2, we outline the framework of modality by Giannakidou & Mari (2018a, b), which we use as the basis for our analysis, and define some predictions made by the system. We proceed with our two experiments in Sects. 3 and 4, followed by discussion and conclusion in Sects. 5 and 6.

## Formal Aspects and Experimental Hypotheses of Bias and Modality

Modal expressions in natural languages are the common devices to reflect the speaker’s epistemic or doxastic stance towards the truth of a proposition. Almost all analyses of modality assume that non-alethic modal expressions are nonveridical (Kratzer, 1977, 1991; Giannakidou, 1997, 1998, 1999, 2013; Condoravdi, 2002; Portner, 2009; Beaver & Frazee, 2016, Giannakidou & Mari, 2016, 2018a, b, 2021a, b, pace von Fintel & Gillies, 2010), i.e., they do not entail that the proposition is true. The function of modal expressions is to convey the nonveridical attitude of the speaker: upon hearing or reading a modal sentence, the audience understands that the speaker cannot be fully, i.e., veridically, committed to the truth of a proposition. This is an important assumption for the experiments we will report in the following sections. For example, consider the following declarative sentences. (8a) uses the present tense, and (8b,c) contain the modal verbs (*must* and *may/might*):

**Table:** 

(8)	a. It is raining.
	b. It must be raining.
	c. It may/might be raining.

Let us call the tensed unmodalized sentences such as (8a) “bare”. In semantics and pragmatics, we assume that in asserting a bare sentence the speaker is saying something that they know or believe to be true—they are, in other words, *veridically* committed to it. Giannakidou & Mari (2018a, b, 2021a, b; see also Giannakidou, 1998, 2013) call this the *veridicality principle of assertion* (and it follows from abiding by Gricean *Quality*, which is fundamental to co-operative conversation). Thus, upon hearing an unmodalized sentence the hearer understands that the speaker knows, or has grounds to believe that it is raining or that it rained. On the other hand, when a speaker chooses to use a modal, they take a *nonveridical stance*, which means that the speaker is uncertain about whether it is raining or not—and this uncertainty is typical also of questions and conditionals. The uncertainty is in nonveridical equilibrium with possibility modals: with *may* or *might* as in (8c), raining is considered a mere possibility, and the speaker has no reason to believe *It is raining* is closer to what is the case than *It is not raining.* Possibility modals are thus very much like conditional protasis and information-seeking questions in this regard.

When a necessity modal such as *must/should* as in (8b) is used, the equilibrium is manipulated towards raining being considered more likely by the speaker. Giannakidou and Mari coin the term ‘biased modals’ to characterize necessity modals: the speaker is *biased* in favor of the prejacent proposition, though they still are not veridically committed to it. Modal bias reveals an epistemic stance supported by evidence in favor of the proposition, but it does not mean that the speaker *knows p* to be true. Modals remain indicators that the speaker reasons with uncertainty and that they leave both options, *p* and *¬p*, open. But with biased modals, the speaker appears to be more committed to* p* than to *¬p*, and this commitment is itself gradient (i.e., *must* expresses more bias toward *p* than *should* or *ought to*, see Portner, 2009 and Sode & Sugawara, 2019 on German *sollte*).

### Formal Aspects

The gradience described above is sometimes captured by positing secondary ordering sources for the modals (Portner, 2009; Portner & Rubinstein, 2016). Giannakidou & Mari (2018a, b, 2021a, b) argue instead that bias is an additional parameter of evaluation for all modals, and it comes in the form of a *ranking metaevalution* function *O.* It is not necessary for the purposes of this paper to go through the entire system, but let us offer the necessary details relevant to conditionals, which will motivate our experimental hypotheses in Sect. 2.2.

MUST associates with an epistemic modal base M(*i*), where *i* is the speaker, which is partitioned by a stereotypical ordering S into Ideal and non-Ideal worlds. Ideal_S_ is a function over M(i)(t_u_)(w_0_), and the output Ideal_S_ is a subset of M(i)(t_u_)(w_0_):

**Table:** 

(9)	M(i)(t_u_)(w_0_) = λw’(w’ is compatible with what is known by the speaker i in w_0_ at t_u_)
(10)	Ideal_S_ (M(i)(t_u_)(w_0_)) = {w’ ∈ M(i)(t_u_)(w_0_): ∀q ∈ S (w’ ∈ q)}

So defined, Ideal_S_ delivers the worlds in the modal base where all the propositions in S are true. S is a set of propositions that corresponds to common ground norms. The truth condition for MUST says that *p* is true in the Ideal set of M(*i*). We give here the truth condition with NONPAST which is the future-shifting tense found in conditionals:

**Table:** 

(11)	Given a set Ideal_S_ and the utterance time t_u_,
	[[must/sollen(NONPAST(p))]]^*M,i,S*^ is defined only if M(*i*) is nonveridical and is partitioned into Ideal_S_ and ¬Ideal_S_ worlds. If defined,
	[[must/sollen(NONPAST(p))]]^M,i,S^ = 1 iff ∀w’ ∈ Ideal_S_: ∃t’ (t’ ∈ ( t_u_, ∞) & p (w’, t’))

The MUST prejacent will be true in Ideal worlds at a future time that includes t_u_ (t_u_, ∞). For more discussion on future shifting and the nonveridicality of MUST, see Giannakidou and Mari for details; for a related analysis of the modalized tense above, see Kaufmann (2005). The key observation here is that only in Ideal worlds is *p* true.

In addition, Ideal worlds are privileged by the ranking function *O* which is an evaluative function that ranks the Ideal worlds as *better possibilities* (in the sense of Portner, 2009 and Kratzer, 1986) producing positive bias. According to Giannakidou and Mari, a modal adverb is typically the realization of *O*:

**Table:** 

(12)	[[∅-Adverb]]^*O,M,i,S*^ = λq. Ideal_S_ is a *better possibility* with respect to ¬Ideal_S_ relative to M(i) and *O* & q
(13)	It **must certainly/definitely** be raining. / It **may possibly** be raining.

When modal verbs and modal adverbs co-occur, such as in (13), the literature sometimes talks about ‘modal concord’ (Geurts & Huitink, 2006; Huitink, 2012, 2014; Grosz, 2010; *a contrario* Anand & Brasoveanu, 2010; Zeijlstra, 2007; Huitink, 2012; Lyons, 1977; Giannakidou & Mari, 2018b). Yalçın (2007: 994) claims that “iterating epistemic possibility operators add no value in the semantics”. In comparison, Giannakidou & Mari (2018b), using the term “modal spread” hold the view that multiple expressions of modality have one semantic role and that the adverb presents the ordering source of the modal. In more detail, the epistemic modal structure involves three ingredients: (1) a nonveridical modal base M(i), (2) a secondary modal base S that partitions M(i) into Ideal_S_ and ¬Ideal_S_ subsets, relying on stereotypical assumptions, and (3) a meta-evaluation *O* triggered by stereotypicality that ranks the Ideal_S_ worlds as better possibilities than ¬Ideal_S_ worlds in M(i). The preference for higher ranking of Ideal_S_ is lexically specified, and MUST and MIGHT differ in their lexical preferences (both use S, but higher ranking of Ideal_S_ is only a feature of MUST). Giannakidou and Mari argue that the adverbs are overt realizations of the meta-evaluation *O*. In the Giannakidou and Mari framework, nonveridical equilibrium can now be rephrased as (14), see (3) in comparison:

**Table:** 

(14)	**Nonveridical equilibrium.** An epistemic state M is in nonveridical equilibrium iff M
	is partitioned into p and ¬p, and *O* is empty.

Non-biased possibility modals, questions and conditionals are in nonveridical equilibrium and have an empty *O*. But if we add modal verbs or adverbs, we see the effect of a non-empty *O*. Here is how Giannakidou (2021) derives REALLY in conditionals.^10^

**Table:** 

(15)	[[REALLY MOD (NONPAST (p))]]^*O,M,i*^ is defined only if
	(i) the modal base M(i) is nonveridical and partitioned into {p, ¬p} worlds, and
	(ii) p worlds are better possibilities than ¬p worlds.

REALLY contributes the definedness condition of *O* for better possibility*,* and we can think of it as a felicity condition attached to the speaker. Because it is a definedness condition of the speaker it can be objected to by the hearer who might not share the bias, and this captures the intuition that bias can be ‘cancelled’ or ‘ignored’, though it is in fact objected to by the hearer’s commitments. Now the propositions are reversed: ¬p worlds are better possibilities, resulting in negative bias. We can understand the positivity in the condition (15-ii) for REALLY as a contextual bias, and the negative bias as the speaker bias, see (16). Both take widest scope, as they project out of conditionals, an entailment-cancelling context (Simons et al., 2010).

**Table:** 

(16)	If Anne really becomes a lawyer, she will open a law firm.
	a. **contextual positive bias:** Anne will become a lawyer.
	b. **speaker negative bias:** It is not the case that Anne will become a lawyer.

The effect of *really* is similar to its effect in questions as in (2c), where the speaker presupposes a contextual positive bias and conveys their negative bias. Giannakidou & Mari (2021b) further argue that the use of an epistemic modal verb in a question indicates increased uncertainty because ∩ O ⊃ M(i), i.e., extending the set of possibilities beyond the modal base (i.e., those worlds considered by the speaker), thereby making it harder to think of what would be a ‘correct’ answer. This accounts for why modal questions seem open-ended and with increased uncertainty. In conditionals, we have both REALLY and modal verbs creating bias—and there may be a detectable difference between increased uncertainty and negative bias, a point to which we return in the discussion of our experiments. Regardless of the precise contribution of each, the fact is that *O* reverses the positivity of REALLY and MUST/*sollte*.

Does *really* reverse the positivity also in declarative sentences? Our intuition says that while its degree modifying use can be in the scope of negation, the bias use cannot, see (17).

**Table:** 

(17)	a. It is **really** (not) raining. / It is *not **really** raining. (polarity-focusing *really*)
	b. It is (not) **really** raining. / It is **really** *not raining. (degree-modifying *really*)

We think bias is observed also in declarative sentences. In a sentence such as *Agnes really passed the exam,* we propose that *really* (a) requires a negative contextual bias (e.g., the speaker’s prior or a contextual salient agent’s attitude) for *¬p* over *p* possibilities, and (b) asserts *p* (creating a speaker positive bias). The requirement (a) is lacking in a sentence without *really.* The oddity of *really* following negation is probably due to scope conflicts between *really* and negation (Liu, 2012). Focusing on nonveridical contexts only, we assume that the negative bias is present not only in questions but also in conditionals, as we illustrated.^11^ Now recall that in the conditional domain, as we showed, not only adverbs but also CCs can convey different commitments towards the antecedent proposition, including the speaker’s doxastic, deontic or emotional evaluation towards the antecedent or the consequent. For example, Visconti (1996) claims that the Italian CC *casomai* ‘if-ever’ (made up of a simple CC *caso* ‘in case, if’ and a NPI *mai* ‘ever’) differs from *nel caso che* ‘in the case that’ in terms of the speaker’s attitude towards the antecedent *p* that is expressed at the level of conventional implicatures: While *nel caso che* is epistemically neutral, *casomai* conveys a negative bias, namely, ‘improbable(p)’. In more recent literature, Liu (2019, 2021) as we mentioned in Sect. 1, have shown that the German CC *falls* expresses a weaker speaker commitment towards the antecedent proposition than *wenn*. In one of the reported experiments using the forced lexical choice task, the author found that the participants chose *falls* significantly more than *wenn* in the context of negative priors, e.g., when the protagonist does not believe the antecedent proposition, see (18) for an example of the used stimuli; a reverse pattern was found in the context of positive priors (see Liu & Wang, 2021 on Mandarin CCs in this regard).

**Table:** 

(18)	*Kathi*	*hat*	*morgen*	*für*	*einen*	*Tag freigenommen.*	*Sie*	
	Kathi	has	tomorrow	for	a	day taken free	she	
	*{glaubt / glaubt nicht}*	*dass*	*es*	*morgen*	*regnet und*			
	believes / believes not	that	it	tomorrow	rains and			
	*denkt:*	*______*	*es morgen*	*regnet,*	*bleibe*	*ich*	*zu*	*Hause.*
	thinks:	______	it tomorrow	rains	stay	I	at	home
	(‘Kathi has taken tomorrow off. She {believes / doesn’t believe} that it will rain tomorrow and is thinking: ______it rains tomorrow, I will stay at home.’)							

### Experimental Hypotheses

In the above, we saw that certain CCs express a weaker speaker commitment than others. Modal verbs such as *must/should* in general express a positive bias (high degree of speaker commitment) towards the modified proposition *p*, i.e., a strong speaker commitment about *p* (but weaker speaker commitment than their unmodalized variant, which is veridical) in declarative sentences (veridicality principle); in nonveridical contexts with nonveridical equilibrium presupposed, we expect that the positive bias towards *p* weakens in comparison to variants without them. In conditionals, the adverb *really* expresses a weaker commitment than the variants without them as well.

In addition to modal verbs, adverbs, and CCs, word order variation within conditionals might also have an effect on distribution and semantics, see the discussion of verb-first (V1) conditionals in comparison to *wenn/falls-*conditionals and questions in German (Reis & Wöllstein, 2010). While we know about the *wenn/falls* contrast in this aspect based on Liu (2019, 2021), it is unclear how they compare with V1-conditionals.

In the rest of the paper, we will deal with multiple expressions of modality in (different kinds of) conditionals and questions in German, with a focus on the adverb *wirklich* ‘really’ and the modal verb *sollte* ‘should’. With regard to their interpretive effects, the theoretical framework of veridicality and bias we have outlined allows us to formulate the hypotheses in (19):

**Table:** 

(19)	Hypotheses of *wirklich* and *sollte* in conditionals and questions
	a. Hypothesis 1. Different sentence types and subtypes convey different degrees of speaker commitment about the modified (antecedent) proposition.
	b. Hypothesis 2. The use of the adverb *wirklich* indicates reduced speaker commitment about the modified (antecedent) proposition.
	c. Hypothesis 3. The use of the modal verb *sollte* indicates reduced speaker commitment about the modified (antecedent) proposition.

We implemented two experiments to test these hypotheses. The two rating experiments in German tested the interpretive effects of sentence types (conditionals vs. questions), conditional types (*wenn* vs. *falls* vs. V1-conditionals), modal verbs (*sollte/würde*) and the adverb *wirklich* ‘really’, which we report in Sects. 3 and 4. We collected subjects’ inference about their belief whether the prejacent *p* is true in different manipulations, based on the assumption that the comprehender’s inference of the speaker’s assumptions reveals the meaning of the used modal expressions. We will discuss the results of both experiments and their implications as well as limitations in Sect. 5.

## Experiment 1

In this experiment, we tested the speaker’s doxastic (i.e., belief-based) commitment in relation to sentence types, the modal verb *sollte* and the adverb *wirklich*. We take the comprehender’s belief judgment to be indicative of speaker (i.e., individual anchor) commitment, and a weaker commitment relates to negative bias. Differing degrees of speaker commitment do not affect the semantic (at-issue) content of the given question or conditional, since, as we said, bias is a precondition on the question or conditional. For example, the high negation such as in *Isn’t Agnes a vegetarian?* in (2a) does not affect the question meaning of the sentence *Is Agnes a vegetarian?* but contributes a speaker meaning at a separate (non-at-issue) dimension.

### Method

#### Participants

83 adult German native speakers (33 female, 50 male, mean age = 30.2, SD = 9.7) participated in the study. The experiment was approved by the ethics committee of the German Linguistic Society.

#### Design and Materials

Experiment 1 was based on a 2 × 2 × 2 factorial design, with three within-participants and within-items factors: SentenceType (*wenn-*conditional vs. *ob-*question) × Adverb (with or without *wirklich* ‘really’) × ModalVerb (with *sollte/würde* ‘should/would’ or without), see an example in (20). We controlled the stimuli in such a way that *wirklich* is used as a polarity-focusing adverb but not as a degree modifier. The reason why we did not use the same modal verb across all the conditions is that *sollte* in embedded questions only gets a deontic or bouletic reading, instead of the doxastic reading which we target. In total, we used 40 target items. Each item consisted of a context-setting sentence (S1), a critical sentence (S2), a critical question (S3) about the speaker’s belief in the prejacent (i.e., the proposition in the conditional antecedent or the question), and a final comprehension question (S4) for attention check. The complete list of target items is included in Appendix 1. In addition, we used 48 filler items of similar structure.

**Table:** 

(20)		S1: *Paula denkt über ihre Zukunft nach.* (Paula is thinking about her future.)
		S2:
	a.	*Sie denkt: „****Wenn**** ich eine Arbeit finde, kaufe ich mir ein Mac Book.“*
		(She thinks to herself: ‘If I find a job, I will buy myself a Mac Book’.)
	b.	*Sie denkt: „****Wenn**** ich ****wirklich**** eine Arbeit finde, kaufe ich mir ein Mac Book.“*
		(She thinks to herself: ‘If I really find a job, I will buy myself a Mac Book’.)
	c.	*Sie denkt: „****Wenn**** ich eine Arbeit finden ****sollte****, kaufe ich mir ein Mac Book.“*
		(She thinks to herself: ‘If I should find a job, I will buy myself a Mac Book’.)
	d.	*Sie denkt: „****Wenn**** ich ****wirklich**** eine Arbeit finden ****sollte****, kaufe ich mir ein Mac Book.“*
		(She thinks to herself: ‘If I should really find a job, I will buy myself a Mac Book’.)
	e.	*Sie fragt sich, ****ob**** sie eine Arbeit findet.* (She asks herself if she will find a job.)
	f.	*Sie fragt sich, ****ob**** sie ****wirklich**** eine Arbeit findet.*
		(She asks herself if she will really find a job.)
	g.	*Sie fragt sich, ****ob**** sie eine Arbeit finden ****würde****.*
		(She asks herself if she would find a job.)
	h.	*Sie fragt sich, ****ob**** sie ****wirklich**** eine Arbeit finden ****würde****.*
		(She asks herself if she would really find a job.)
		S3: *Glaubt Paula, dass sie eine Arbeit findet?*
		(Does Paula believe that she will find a job?)
		S4: *Denkt Paula über Ihre Oma nach?*
		(Is Paula thinking about her grandmother?)

 While “nonveridical equilibrium” is assumed to be the default for both conditionals and questions in Giannakidou & Mari (2018a, b, 2021a, b), it is to note that *wenn*-sentences in German are ambiguous between temporal and conditional interpretations. In relation to this duality, it is feasible to assume that *wenn-*conditionals may carry a positivity that embedded *ob-*questions do not. Based on this and the literature we outlined above, we formulated the following specific predictions for Experiment 1.

E1.P1 (see 19a): *Ob-*questions would receive lower ratings of speaker commitment than *wenn*-conditionals (i.e., concerning the antecedent proposition). We thus expected an effect of SentenceType.

E1.P2 (see 19c): The adverb *wirklich* would lower the ratings of speaker commitment in both questions and conditionals. We thus expect an effect of the adverb in the overall data as well as in the sub-analysis of the *wenn*-conditional or the *ob*-question dataset.

E1.P3 (see 19d): The modal verb *sollte* would lower the ratings of commitment. We thus expected an effect of the modal verb in the sub-analysis of the *wenn*-conditional conditions. We did not have specific predictions for the verb *würde*, but as it is strictly speaking not a modal verb, we expected it to be different from *sollte*.

#### Procedure

Participants took part in the study at the online crowd-sourcing platform Prolific (https://www.prolific.co/) for small payments. They started with four practice trials. For the target items, they read S1 and S2, and then answered the question in S3 on a 7-point Likert scale (with labelled endpoints, i.e., 1 = *Stimmt gar nicht* ‘absolutely no’, 7 = *Stimmt vollkommen* ‘absolutely yes’), which we take to reflect the degree of the speaker or the doxastic agent’s, e.g., Paula’s commitment in (20), to the given proposition in S2. They also answered the polar question in S4 with “yes” or “no”. The experiment was programmed and hosted on Ibex Farm (Drummond, 2013). Each participant saw all 40 target items and 48 filler items presented in a pseudorandom order. The total experimental duration was approximately 25 min.

#### Data Analysis

First, we assessed participants’ response accuracy on the comprehension questions. The data of one participant with a response accuracy below 85% was ruled out. In total, the data of 82 participants were analyzed.

We analyzed the rating data via cumulative link mixed models for ordinal regression implemented in the ordinal package (Christensen, 2019) in R (R Core Team, 2019). We conducted an analysis of the entire data set. In addition, we also conducted two separate analyses of Adverb and ModalVerb in the *wenn-*conditional type and the *ob-*question type. For all models, the factors were manually sum coded with ± 0.5, that is, SentenceType (*wenn-*conditional 0.5, *ob-*question − 0.5) × Adverb (without *wirklich* ‘really’ 0.5, or with it − 0.5)× ModalVerb (without *sollte/würde* ‘should/would’ 0.5, or without it − 0.5). The cumulative link mixed model included the logit-link function. We used the most parsimonious model approach (Bates et al, 2015): The model including random by-subject and by-item intercepts was chosen as it fit the data best.^12^ The *p*-values were obtained through model comparison via likelihood ratio tests between the model without the respective effect against the full model (Christensen, 2019). In the case of significant effects, we provide the *p*-values rounded to three decimals unless they are smaller than 0.01; in the case of non-significant effects, *p*-values are rounded to two decimals. We report the results below.

### Results

**Fig. 1:**
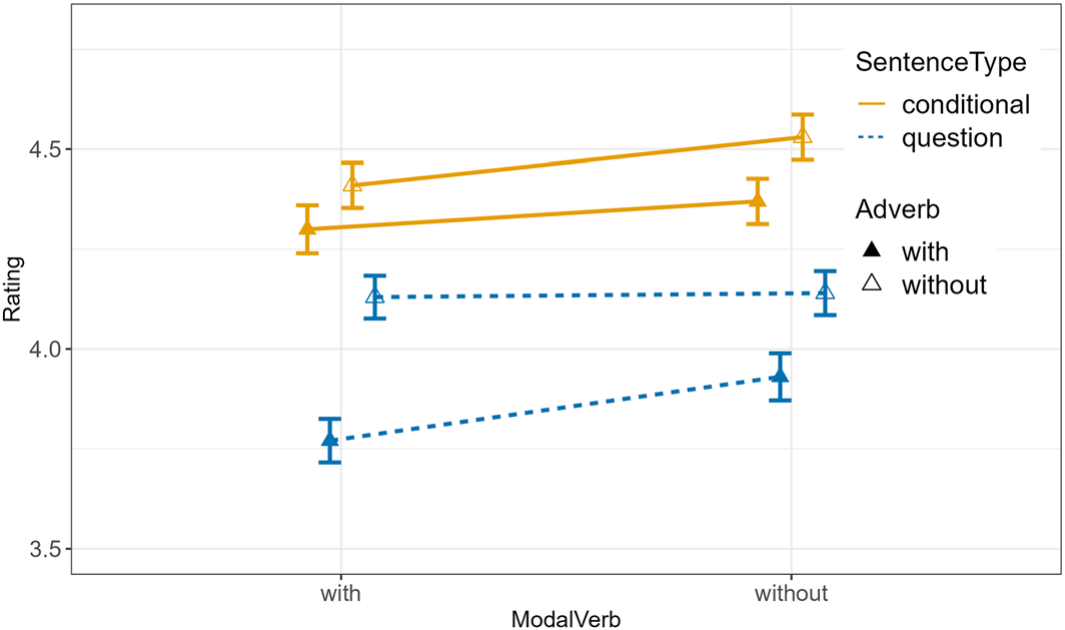
Means and error bars of speaker commitment ratings (7-point Likert scale, 1 = absolutely no, 7 = absolutely yes) for the eight conditions of Experiment 1. The bars connected with solid lines are for *wenn*-conditionals. The bars connected with dashed lines are for *ob*-questions. Bars with filled vs. empty triangles are for conditions with vs. without *wirklich*. The conditions with vs. without modal adverbs are plotted on the left vs. right side of the figure (color figure online)

The rating responses are visualized in Fig. 1, with the descriptive statistics in Table 1.

**Table 1:** Participants’ mean ratings on a 1–7 Likert scale for the eight conditions of Experiment 1

Condition	SentenceType (*wenn* vs. Q)	Adverb (*wirklich*)	ModalVerb (*sollte/würde*)	S3-ratings (standard deviation)
1	wenn	*–*	*–*	4.52 (1.15)
2	wenn	+	*–*	4.36 (1.15)
3	wenn	*–*	+	4.40 (1.14)
4	wenn	+	+	4.30 (1.22)
5	Q	*–*	*–*	4.12 (1.12)
6	Q	+	*–*	3.93 (1.21)
7	Q	*–*	+	4.12 (1.09)
8	Q	+	+	3.77 (1.11)

The ‘–’ indicates the absence of the given expression and the ‘ + ’ sign its presence. Mean and standard deviation are rounded to the second decimal

The results of the entire data set (see Table 2) are the following: (1) There was a significant effect of SentenceType with lower ratings for the *ob*-question conditions than for the *wenn-*conditional conditions ($\widehat{\upbeta }$=0.82, *p* < 0.0001), confirming E1.P1. (2) There was a significant effect of Adverb with lower ratings for the conditions with *wirklich* than without *wirklich* ($\widehat{\upbeta }$=0.43, *p* < 0.0001), confirming E1.P2. (3) There was a significant effect of ModalVerb in general with lower ratings for the conditions with ModalVerb than without it ($\widehat{\upbeta }$=0.18, *p* = 0.007).

**Table 2:** Output of Analysis for Experiment 1 using cumulative link mixed models with subject and item as random effects

Fixed effects				Model comparison		
Estimate	$\widehat{\upbeta }$	Std. Error	z-value	χ^2^	DF	*p*-value
SentenceType	0.82	0.07	12.13	150.92	1	< 0.0001
Adverb	0.43	0.07	6.56	43.31	1	< 0.0001
ModalVerb	0.18	0.07	2.69	7.23	1	0.007
Interaction: SentenceType:Adverb	− 0.16	0.07	− 2.38	5.66	1	0.02
Interaction: SentenceType:ModalVerb	0.02	0.07	0.27	0.07	1	0.79
Interaction: Adverb:ModalVerb	− 0.06	0.07	− 0.95	0.91	1	0.34
Interaction: SentenceType:Adverb:ModalVerb	0.08	0.07	1.28	1.63	1	0.20

formula = clmm(Rating ~ SentenceType + Adverb + ModalVerb + SentenceType:Adverb + SentenceType:ModalVerb + Adverb:ModalVerb + SentenceType:Adverb:ModalVerb + (1|subj) + (1|item)

While we did not have specific predictions about the interaction of the tested effects, the model shows that the three-way interaction between SentenceType × Adverb × ModalVerb was not significant. The two-way interactions between SentenceType × ModalVerb or Adverb × ModalVerb were not significant either, but the two-way interaction between SentenceType × Adverb was significant. Given the significant SentenceType × Adverb interaction, we conducted two sub-analyses looking into the effect of Adverb in questions and conditionals separately. We included the factor of ModalVerb in the model despite the lack of the SentenceType × ModalVerb interaction to further explore the data. The results show that the effect of Adverb was greater in questions ($\widehat{\upbeta }$=0.69, *p* < 0.0001) than in conditionals ($\widehat{\upbeta }$=0.28, *p* = 0.003), explaining the interaction effect. The effect of *sollte* in *wenn-*conditionals ($\widehat{\upbeta }$=0.21, *p* = 0.03) was slightly different from the effect of *würde* in *ob*-questions ($\widehat{\upbeta }$=0.17, *p* = 0.07), explaining the lack of interaction. In the rest of the paper, we will only deal with *sollte* in conditionals.

## Experiment 2

In Experiment 1, we tested the *wenn-*conditionals in comparison to *ob*-questions, with the finding that the questions convey lower speaker commitment to the modified proposition than conditionals. Furthermore, we found a commitment-weakening effect of the adverb *wirklich* across both sentence types, as well as a similar effect of the modal verb *sollte* in conditionals.

We decided to look closely at bias and modality in conditionals in a follow-up study, in particular, because different kinds of conditionals have been argued to differ in semantics and pragmatics, and thus might convey different degrees of speaker commitment: Among others, Liu (2019, 2021), for example, provide distributional and experimental evidence that *falls-*conditionals convey lower speaker commitment to the antecedent proposition than *wenn*-conditionals. Reis & Wöllstein (2010), as we introduced earlier in the paper, suggest that V1-conditionals might be semantically related to questions and that they are different from *wenn-*conditionals. In order to examine whether the effects of *wirklich* and *sollte* we found in Experiment 1 obtain across different types of conditionals (*wenn/falls/*V1-conditionals) and how they interact with one another, we conducted a second experiment of an explorative nature. It is meant as a first step that might lead to hypotheses for further testing in a more strict and comprehensive way.

We considered three types of indicative conditionals in German, one with the CC *wenn*, the other with the CC *falls*, which has been argued to indicate a lower degree of commitment (Liu, 2019). The third type are the so-called V1-conditionals (with the finite verb appearing in the initial position of the antecedent clause) which Reis & Wöllstein (2010), among others, discuss. We will not discuss the formal aspects of these expressions in detail but only the following aspects related to the experiment we conducted.

First, it has been noted that in the case of hypothetical use of *falls,* it often co-occurs with the modal verb *sollte*, such as in (21)*.* Second, in V1-conditionals in the hypothetical use, *sollte* is often used as well, such as in (22). This contrasts with the strong necessity verbs *muss/müsste* which cannot be used here, see Sode & Sugawara (2019) and the corpus findings of Hacquard & Wellwood (2012) for the English strong necessity verb *must*, which in conditionals almost exclusively has root interpretations instead of epistemic ones.

**Table:** 

(21)	*Auf diese Weise wird verhindert, daß explosive Gasgemische entstehen, ****falls**** der* *Gasbehälter einmal ein Leck haben ****sollte****.* (Bild der Wissenschaft, 2/1967, 146) 13
	‘In this way it is prevented that explosive gas mixtures arise if the gas container should ever leak.’
(22)	***Sollte**** er es aber nicht wissen, so werde ich mich bemühen, ihm die deutsche*
	*Friedfertigkeit klarzumachen.* (Bild, 8.3.1967, 4) 14
	‘But, should he not know it, I will then try to make the German peacefulness clear to him.’

Reis & Wöllstein (2010) argue that V1-conditionals (as well as *falls-*conditionals) are different from *wenn-*conditionals, and based on distributional facts, suggest tentatively that V1-conditionals and questions might have a common semantic core. While their focus is on the structural properties of V1-conditionals, in Experiment 2, we tested these three conditional types and their interaction with *sollte* and *wirklich* focusing on the interpretive effects with regard to speaker commitment about the antecedent proposition.

### Method

#### Participants

89 adult German native speakers (32 female, 57 male, mean age = 29.4, SD = 8.4) participated in the study. The experiment was approved by the ethics committee of the German Linguistic Society.

#### Design and Materials

Experiment 2 was based on a 2 × 2 × 2 factorial design, with three within-participants and within-items factors: ConditionalType (*falls-*conditional vs. V1-conditional), Adverb (with or without *wirklich*) and ModalVerb (with or without *sollte*), see an example in (23). In addition, we also used Condition 1 from Experiment 1 with *wenn* as a control condition. In total, we used 45 target items, with 40 from Experiment 1 and 5 new items in order to create counter-balanced sets; the complete list of target items is included in Appendix 1. As in Experiment 1, each item consisted of a context-setting sentence (S1), a critical sentence (S2) and a critical question (S3) about the speaker’s belief in the antecedent proposition in S2 and a final comprehension question (S4) for attention check. In addition, we used the same 48 filler items as in Experiment 1.

**Table:** 

(23)		S1: *Paula denkt über ihre Zukunft nach.* (Paula is thinking about her future.)
		S2:
	a.	*Sie denkt: „****Wenn**** ich eine Arbeit finde, kaufe ich mir ein Mac Book.“*
		(She thinks to herself, ‘If I find a job, I will buy myself a Mac Book.’)
	b.	*Sie denkt: „****Falls**** ich eine Arbeit finde, kaufe ich mir ein Mac Book.“*
		(She thinks to herself, ‘In case I find a job, I will buy myself a Mac Book.’)
	c.	*Sie denkt: „****Falls**** ich ****wirklich**** eine Arbeit finde, kaufe ich mir ein Mac Book.“*
		(She thinks to herself, ‘In case I really find a job, I will buy myself a Mac Book.’)
	d.	*Sie denkt: „****Falls**** ich eine Arbeit finden ****sollte****, kaufe ich mir ein Mac Book.“*
		(She thinks to herself, ‘In case I should find a job, I will buy myself a Mac Book.’)
	e.	*Sie denkt: „****Falls**** ich ****wirklich**** eine Arbeit finden ****sollte****, kaufe ich mir ein Mac Book.“*
		(She thinks to herself, ‘In case I should really find a job, I will buy myself a Mac Book.’)
	f.	*Sie denkt: „****Finde**** ich eine Arbeit, kaufe ich mir ein Mac Book.“*
		(She thinks to herself, ‘If I find a job, I will buy myself a Mac Book.’)
	g.	*Sie denkt: „****Finde**** ich ****wirklich**** eine Arbeit, kaufe ich mir ein Mac Book.“*
		(She thinks to herself, ‘If I really find a job, I will buy myself a Mac Book.’)
	h.	*Sie denkt: „****Sollte**** ich eine Arbeit finden, kaufe ich mir ein Mac Book.“*
		(She thinks to herself, ‘Should I find a job, I will buy myself a Mac Book.’)
	i.	*Sie denkt: „****Sollte**** ich ****wirklich**** eine Arbeit finden, kaufe ich mir ein Mac Book.“*
		(She thinks to herself, ‘Should I really find a job, I will buy myself a Mac Book.’)
		S3: *Glaubt Paula, dass sie eine Arbeit findet?*
		(Does Paula believe that she will find a job?)
		S4: *Denkt Paula über Ihre Oma nach?*
		(Is Paula thinking about her grandmother?)

The literature we reviewed above only allows to make predictions about main effects, see (19), thus, we formulated the following specific predictions for Experiment 2.

E2.P1 (see 19b): Different kinds of conditionals would receive different speaker commitment ratings. Thus, we expected a difference between the *wenn-* vs. *falls*-conditional or V1-conditional conditions (i.e., Condition 1 vs. 2/6 in Table 3 below) in that *falls-*conditionals would receive lower speaker commitment ratings than *wenn-*conditionals, and possibly V1-conditionals would also receive lower speaker commitment ratings than *wenn-*conditionals. We did not have clear predictions about an effect of ConditionalType (*falls*-conditionals vs. V1-conditionals, i.e., Condition 2–9 in Table 3 below).

**Table 3:** Participants’ mean ratings on a 1–7 Likert scale for the nine conditions of Experiment 2

Condition	SentenceType (wenn vs. Q)	Adverb (wirklich)	ModalVerb (sollte/würde)	S3-ratings (standard deviation)
1	wenn	–	–	4.44 (1.18)
2	falls	–	–	4.30 (1.12)
3	falls	+	–	4.12 (1.24)
4	falls	–	+	4.24 (1.15)
5	falls	+	+	4.12 (1.23)
6	V1	–	–	4.44 (1.10)
7	V1	+	–	4.25 (1.29)
8	V1	–	+	4.24 (1.12)
9	V1	+	+	4.18 (1.28)

The ‘–’ indicates the absence of the given expression and the ‘ + ’ sign its presence

E2.P2 (see 19c): The adverb *wirklich* would lower the ratings of speaker commitment across different kinds of conditionals. We thus expected an effect of the adverb in the overall data as well as in the sub-analysis of the *falls*-conditional or the V1-conditional dataset.

E2.P3 (see 19d): The modal verb *sollte* would lower the ratings of speaker commitment across different kinds of conditionals. We thus expected an effect of the modal verb in the overall data as well as in the sub-analysis of the *falls*-conditional or the V1-conditional dataset.

#### Procedure

The procedure of Experiment 2 was identical to Experiment 1. Participants took part in the study online at Prolific for small payments. They started with four practice trials. For the target items, they read S1 and S2, and then answered the question in S3 on a 7-point Likert scale (with labelled endpoints, i.e., 1 = *Stimmt gar nicht* ‘absolutely no’, 7 = *Stimmt vollkommen* ‘absolutely yes’), which we take to reflect the degree of the doxastic agent’s commitment to the given proposition in S2. They also answered the polar question in S4. The experiment was programmed and hosted on Ibex Farm (Drummond, 2013). Each participant saw all 45 target items and 48 filler items presented in a pseudorandom order. The total experimental duration was approximately 30 min.

#### Data Analysis

First, we assessed participants’ response accuracy on the comprehension questions. Four participants had a response accuracy < 85%, and their data were not included in the analysis.

We analyzed the rating data via cumulative link mixed models for ordinal regression implemented in the ordinal package (Christensen, 2019) in R (Core Team, 2019). First, we conducted a one-factorial analysis of Conditions 1, 2 and 6 (see Table 3 and Analysis 2a below), which correspond to the three conditional types without *wirklich* or *sollte*. For these, we used treatment coding with *wenn* as reference level plus a slope for *falls* and another slope for V1. Second, we conducted an analysis of Conditions 2–9 (Analysis 2b) based on a 2×2×2 factorial (i.e., ConditionalType×Adverb×ModalVerb) within-participants and within-items design. In the model, the factors were manually sum coded with ± 0.5, i.e., ConditionalType (V1*-*conditional 0.5 vs. *falls*-conditional -0.5), Adverb (without *wirklich* ‘really’ 0.5 or with it − 0.5) and ModalVerb (without *sollte* ‘should’ 0.5 or with it -0.5). The cumulative link mixed model included the logit-link function. We used the most parsimonious model approach (Bates et al., 2015) and added random by-subject or by-item intercepts for the effects, or both if possible (i.e., when the better fitting model converged).^15^ The *p*-values were obtained through model comparison via likelihood ratio tests between the model without the respective effect against the full model (Christensen, 2019). They are reported as in Experiment 1. We report the results below.

### Results

**Fig. 2:**
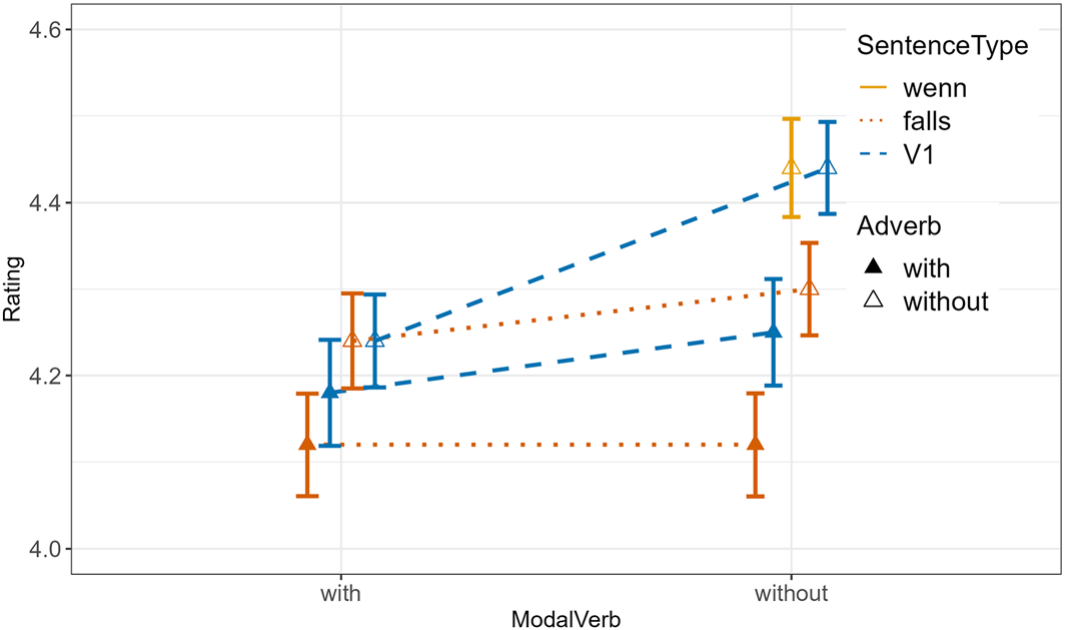
Means and error bars of speaker commitment ratings (7-point Likert scale, 1 = absolutely no, 7 = absolutely yes) for the nine conditions of Experiment 2. The single bar without any connecting line is for *wenn*-conditionals. The bars connected with dotted lines are for *falls*-conditionals. The bars connected with dashed lines are for V1-conditionals. Bars with filled vs. empty triangles are for conditions with vs. without *wirklich*. The conditions with versus without *sollte* are plotted on the left vs. right side of the figure (color figure online)

The rating responses are visualized in Fig. 2, with the descriptive statistics in Table 3.

#### Analysis 2a

The results of the one-factorial analysis of Condition 1, 2 and 6 (see Table 4) are the following. (1) The difference between Condition 2 vs. 1 (*falls* vs. *wenn*) was significant with lower ratings in the *falls-*condition than in the *wenn*-condition ($\widehat{\beta }$=-0.34, *p* = 0.01). (2) The difference between Condition 6 vs. 1 (V1 vs. *wenn*) was not significant.

**Table 4:** Output of Analysis 2a of Experiment 2 using cumulative link mixed models with subject and item as random effects; formula = clmm(Rating ~ falls + v1 + (1|subj) + (1|item)

Fixed effects				Model comparison		
Estimate	$\widehat{\upbeta }$	Std. Error	z-value	χ^2^	DF	*p*-value
*falls*	− 0.34	0.13	− 2.51	6.32	1	0.01
V1	0.04	0.13	0.32	0.1	1	0.75

#### Analysis 2b

The results of the analysis for Condition 2–9 based on a 2 × 2 × 2 factorial (i.e., ConditionalType×Adverb×ModalVerb) design (see Table 5) are the following. (1) There was a significant effect of ConditionalType, with lower ratings for *falls*-conditionals than for V1*-*conditionals ($\widehat{\upbeta }$=0.16, *p* = 0.013). (2) There was a significant effect of Adverb with lower ratings for conditions with *wirklich* than without it ($\widehat{\upbeta }$=0.25, *p* < 0.0001). 4) There was a significant effect of ModalVerb with lower ratings for conditions with *sollte* than without it ($\widehat{\upbeta }$=0.16, *p* = 0.02). (3) There was no three-way interaction or any two-way interactions.

**Table 5:** Output of Analysis 2b of Experiment 2 using cumulative link mixed models with subject and item as random effects

Fixed effects				Model comparison		
Estimate	$\widehat{\upbeta }$	Std. Error	z-value	χ^2^	DF	p-value
ConditionalType	0.16	0.06	2.5	6.23	1	0.01
Adverb	0.25	0.06	3.93	15.48	1	< 0.0001
ModalVerb	0.16	0.06	2.44	5.97	1	0.02
Interaction: ConditionalType:Adverb	0	0.06	0.01	0	1	0.99
Interaction: ConditionalType:ModalVerb	− 0.1	0.06	− 1.58	2.51	1	0.11
Interaction: Adverb:ModalVerb	0.07	0.06	1.09	1.19	1	0.28
Interaction: ConditionalType:Adverb:ModalVerb	− 0.05	0.06	− 0.84	0.7	1	0.40

formula = clmm(Rating ~ ConditionalType + Adverb + ModalVerb + ConditionalType:Adverb + ConditionalType:ModalVerb + Adverb:ModalVerb + ConditionalType:Adverb:ModalVerb + (1|subj))

## Discussion

In the current study, we tested degrees of doxastic commitment about a given proposition as indication of speaker bias. The observed effects are a result of diverse manipulating sources having to do with modality: sentence types (*wenn*-conditionals vs. *ob*-questions) and subtypes (*wenn/falls/*V1-conditionals), the modal verbs (*sollte*) and the adverb (*wirklich*). The results are summarized in Table 6 and the list of (24).

**Table:** 

(24)	Comparison of the results to the hypotheses in (19):
	a. Hypothesis 1 was confirmed: Different sentence types and subtypes convey different degrees of speaker commitment about the modified (antecedent) proposition.
	b. Hypothesis 2 was confirmed. The use of the adverb *wirklich* indicates reduced speaker commitment about the modified (antecedent) proposition.
	c. Hypothesis 3 was confirmed. The use of the modal verb *sollte* indicates reduced speaker commitment about the modified (antecedent) proposition.

**Table 6:** Summary of results of Experiment 1 and Experiment 2

Experiment 1		Experiment 2	
Sentence types	*ob*-question < *wenn*	Conditional types	*falls* < wenn/V1
Adverb	with < without	Adverb	with < without
ModalVerb	with < without	ModalVerb	with < without

The symbol < indicates a statistically significant difference with the former getting lower ratings than the latter

In the following, we will discuss the results concerning the factors one by one.

### Sentence Types and Subtypes

In Experiment 1, we found a significant effect of SentenceType, with higher ratings for the *wenn*-conditional conditions than for the *ob-*question conditions. Our results show a higher rating in *wenn-*conditionals with mean ratings of 4.44 on a 7-point scale, and 4.57 for Condition 1 without *sollte* or *wirklich* in the antecedent; *ob-*questions have mean ratings of 4.00 on a 7-point scale, and 4.22 for Condition 5 without *sollte* or *wirklich*, so equal to or slightly above the median 4. If we relate “nonveridical equilibrium” (Giannakidou & Mari, 2018a, b, 2021a, b) to the median, the reason for the higher rating with *wenn-*conditionals might lie in the fact that asking a question places the speaker in a de facto position of informational neutrality while in the conditional protasis speaker and comprehender treat *p* as a *condition* for the antecedent to be true, hence as a ‘given’ or even a cause for the consequent to happen. Thus, while both questions and conditional protases are logically in a nonveridical equilibrium, the equilibrium is masked by the contentful relation between antecedent and consequent, while questions are monoclasual and the issue does not arise. Additionally and more importantly, as we mentioned in Sect. 2, we must note that the CC *wenn* in German is also used as a temporal connective, such as in the ambiguous example in (25). In relation to this duality, it is feasible to assume that *wenn-*conditionals may carry a positivity that embedded questions do not, in line with the experimental results.

**Table:** 

(25)	Wenn	wir	das	Spiel	gewinnen,	verraten	sie	uns	ihr	nächstes	Ziel.^16^
	if/when	we	the	game	win	tell	they	us	their	next	goal
	‘If/When we win the game, they'll give us the next goal.’										

For this reason, we took a closer look at the stimuli, which reveals that some of the *wenn*-sentences are ambiguous between a temporal and a conditional reading.^17^ For example, the test item (1), stated in (26a), is ambiguous between a temporal and a conditional reading, but the item (3), stated in (26b), can only have a conditional reading, see Appendix 1.

**Table:** 

(26)	a.	*Wenn*	*ich*	*Anwältin*	*werde,*	*mache*	*ich*	*eine*	*eigene*	*Anwaltskanzlei*		*auf.*
		if/when	I	lawyer	become	open	I	an	own	law firm		on
		‘If/When I become I lawyer, I will open a law firm of my own.’										
	b.	*Wenn*	*Mario*	*meine*	*Jacke*	*dabei*	*hat,*	*kann*	*ich*	*sie*	*anziehen.*	
		if	Mario	my	jacket	with	has,	can	I	it	wear	
		‘If Mario has my jacket with him, I can put it on.’										

In this regard, world knowledge plays a role too: most careers will probably see at least one promotion, so the temporal reading of the item (7) in the stimuli, stated in (27a), is quite easily accommodated. In comparison, it is certainly possible for a football team to never win a championship, so a temporal reading for the item (9), stated in (27b), requires enriching the context with the assumption that the protagnist’s team is good.

**Table:** 

(27)	a.	*Wenn*	*ich*	*eine*	*Beförderung*	*bekomme,*	*mache*	*ich*	*eine*	*Feier.*		
		if/when	I	a	promotion	get	make	I	a	party		
		‘If/When I get a promotion, I will have a party.’										
	b.	*Wenn*	*meine*	*Mannschaft*	*die*	*Meisterschaft*	*gewinnt,*	*rasiere*	*ich*	*meine*	*Haare*	*ab.*
		if/when	my	team	the	championship	wins	shave	I	my	hair	off
		‘If/When my team wins the championship, I will shave my hair off.’										

Thus, it is worthwhile to systematically check for the availability of temporal readings in the materials in follow-up studies. However, we need to be cautious with claiming that the ambiguity is the (sole) reason for the difference between *ob*-questions vs. *wenn*-conditionals, as in Experiment 2 we did not find a difference between *wenn*-conditionals and V1-conditionals even though the latter do not have a temporal reading.

Our experimental results also raise the question whether *wenn* is the default CC in German. While *wenn* is the most frequent CC in German, researchers do not have a consensus regarding the question whether *wenn* or *falls* is the prototypical CC (Breindl et al., 2014). It is further worth noting that in our stimuli we used embedded *ob-*questions, which might differ from default unembedded questions such as *Sie denkt: „Finde ich eine Arbeit?”* or *Sie denkt: „Ob ich eine Arbeit finde?”* (She thinks to herself: Will I find a job?). We will leave the last point for future investigation as well.

In Experiment 2, we found a significant difference for the *wenn*-conditionals vs. the *falls*-conditionals in that the latter received lower ratings in line with our predictions based on Liu (2021). In addition, the *falls*-conditionals received lower ratings than the V1-conditionals which did not differ from *wenn-*conditionals. While nonveridical equilibrium is assumed to be the default for conditionals, our results also show variation between different conditional types. The interpretive effect of V1-conditionals in comparison to the others (i.e., similar to *wenn*, but different from *falls*) has not been previously elicited experimentally, to our knowledge, and this is relevant for our understanding of V1-conditionals in general. For example, Reis & Wöllstein (2010), focusing on morphosyntactic questions of conditionals, argue that V1-conditionals (as well as *falls-*conditionals) are different from *wenn-*conditionals, but our data provides a different picture from a pragmatic perspective. While the *wenn* vs. *falls* contrast is oftentimes attributed to the temporal interpretation of *wenn* which *falls* does not have, the lack of a difference between *wenn* vs. V1-conditionals with no temporal reading casts doubt on the idea that temporality is the sole explanation for the *wenn* vs. *falls* contrast.

### The Adverb *wirklich*

In both experiments, we found a significant effect of *wirklich* in that conditions with it received lower ratings than without it, showing a weakening effect of doxastic commitment by the speaker, as understood by the comprehender. This holds across different sentence or conditional types, which was consistent with our predictions. There are several aspects in the results that we will discuss here briefly.

First, we did not find any interaction effects in both experiments, except for a significant two-way interaction between SentenceType x Adverb in Experiment 1 in that the effect of Adverb was greater in questions than in conditionals, that is, *wirklich* lowered the ratings to a greater extent in questions than in conditionals. We do not have a definite explanation for this, but note that questions (P?) and conditionals (If P, Q) address different questions under discussion (QUDs)—the former about P, and the latter relating to two different kinds of QUDs, namely “Under which conditions Q?” and “What follows from P?”, see Fintel (2001) and Arregui & Biezma (2016). The question “*Wirklich* P?” address the same QUD as one without *wirklich*. On the other hand, conditionals with *wirklich* seem to relate to only the QUD “What follows from P?”, that is, with Q being the more salient proposition at-issue than P, unlike in questions. This might explain the SentenceType x Adverb interaction in Experiment 1 and the absence of an interaction effect in Experiment 2 as the latter only tested conditionals.

A reviewer suggested to us that *wirklich* is anaphoric—an observation consistent with our analysis of REALLY mentioned earlier. Recall from earlier discussion that for REALLY we proposed a contextual and a speaker bias, repeated in (28).

**Table:** 

(28)	If Anne really becomes a lawyer, she will open a law firm.
	a. **contextual positive bias:** Anne will become a lawyer.
	b. **speaker negative bias:** It is not the case that Anne will become a lawyer.

The contextual bias of REALLY, by the speaker and hearer via their previous mental state in the context, renders it anaphoric, and the speaker bias produces the negativity. REALLY is thus different form a purely modal element such as *sollte*, which lacks the contextual bias. In this regard, REALLY is also distinct from *falls*, which is not anaphoric either. This difference needs to be considered in future studies targeting the interaction between these expressions (e.g., in relation to modal spread).

A reviewer also pointed out to us that the presence of *wirklich* in *wenn*-conditionals makes a temporal reading impossible. We agree with this intuition; however, we do not think this is the reason for the effect of *wirklich*, as its effect in questions in Experiment 1 was greater, and also because there was an effect of *wirklich* in the *falls-* and V1-conditionals in Experiment 2, even though neither type of conditionals has a temporal interpretation.

On an additional note, the future semantic investigation of *wirklich* (*really/truly*) might benefit from including their close relatives such as *tatsächlich* (*actually/indeed*)*.* While they look similar at first sight, there are distributional differences that hint at potential differences: for example, all the native speakers we consulted share the intuition that the effect of *tatsächlich* ‘in fact, actually’ in conditionals is comparable to that of *wirklich*, but it has been pointed out to us (Manfred Krifka, p.c.) that while *tatsächlich* can occur in the pre-field in German, *wirklich* cannot.

### The Modal Verb *sollte*

In both experiments, we found a significant effect of *sollte* in that conditions without it received higher ratings than with it, showing that it has a weakening effect in terms of speaker commitment.

As an alternative to the “weakened commitment” analysis, Sode & Sugawara (2019), argue that both *sollte*, in their term “on its deliberative use” in contrast to weak necessity modals, and *falls* introduce a use condition that takes the truth of the antecendent proposition *p* as “a truly open possibility against a given conversational background” (see Reis & Wöllstein, 2010 for a similar point, as we presented in Sect. 1). By this, they stand in contrast to V1-/*wenn*-conditionals, which we can then take to be positively biased. Either perspectives are in line with the lower ratings with *sollte* in our experimental data.

A related aspect we plan to investigate in future studies concerns the effect of *sollte* (and possibly also *wirklich*) in *falls*-conditionals. Since both *falls* and *sollte* express negative bias towards the antecedent proposition as our studies show, and they often co-occur (Reis & Wöllstein, 2010), the question is whether they work as one modality structure, as expected by the Giannakidou and Mari framework of modal spread we adopted.

### The Nature of Bias

We provided experimental evidence for bias triggered by modal devices, and in closing, we want to offer some more comments on the nature of bias as it emerges from our findings and hypotheses. Bias, as we said, is *individually* anchored, i.e., to the speaker or comprehender who makes assumptions about the speaker’s belief state. As we said at the beginning, bias goes in different directions (either positive or negative) and is itself a doxastic state, i.e., the belief or credence of the individual anchor, prior to asserting the conditional or asking a question, that the proposition will play out in the positive or negative direction. This credence is the result of all the factors that form belief, i.e., the anchor’s knowledge, beliefs, expectations plus contextual biases that are given by the context.

So, what is the semantic or pragmatic status of bias? Is it a presupposition, an implicature, or a felicity condition? One must admit that the bias belongs to the category of *non-at-issue* content, and is speaker-oriented (Liu, 2012; Potts, 2005), except for the contextual bias in the case of *wirklich*, which is not always anchored to the speaker. We have suggested, following Giannakidou (2021), that speaker bias is best understood as a felicity condition attached to the speaker, e.g., like specificity conditions (Ionin, 2006, Giannakidou & Quer, 2013). Felicity conditions are definedeness conditions that are *not* motivated in the common ground. We might think of them as being similar to conventional implicatures in the sense of Potts (2005) or even weaker, as lexically triggered conversational implicatures. 

In the course of the discussion, we have been characterising bias both as “weakened commitment” and “increased uncertainty” of the anchor. While we used explicitly the anchor’s belief (or credence) as the measurement in both experiments, it would be interesting to see whether testing on uncertainty will yield similar or different results—by, for example, asking subjects how certain Paula is that she will find a job. However, the certainty measure can probably work well for conditionals but it might not work for questions, or at least might result in very low ratings for them. This is a methodological question worth further investigation.

An open question concerns the strength of the bias. There are likely nontrivial differences (effects of gradience) between the bias-creating devices we mentioned in the paper: high negation, negative tag, NPI, modal verbs, adverbs, conditional connectives. We are, unfortunately, not able to address the differences in the present study, but we hope to have offered a solid rationale for how to address bias experimentally that can be used to design similar experiments with other devices. It is to be expected, we think, that, just like with modals, the properties of the different classes of expressions might affect the manifestation or degree of bias. For instance, the bias of minimizer NPIs (such as *Does he give a damn about what I say?*) is distinctively stronger than that of a simple NPI *Have you ever been to London?* In addition, the bias in the latter might be optional (that is, absent in some contexts, e.g., in the sentence *Have you ever been to London, by any chance?*). More empirical study is needed to establish such patterns in conditionals.

Finally, another open question concerns the interaction between the broad discourse context and the local sentence context where bias-triggering expressions occur. For example, a reviewer pointed out to us that S1 in the experiments is differently biased with respect to the target conditional between items: doing a marathon training (item (2), see Appendix) usually culminates in running a marathon, i.e., being able to clear the distance, so this item seems positively biased. Conversely, someone who often has to work late (item (6), see Appendix) will probably not have Friday evening off without any further evidence, so this item seems negatively biased. We checked the ratings on these items: Item (2) received the overall rating of 4.41 and item (6) 4.09, which is in line with the reviewer’s comment. On the other hand, a closer look at e.g., the respective ratings in Condition 1 (the conditional condition without *wirklich* or *sollte*) show an opposite pattern with 4.78 for item (2) and 5.10 for item (6). Thus, we will leave a systematic check for S1 bias and subsequent item analysis for future work.

## Conclusion

In this paper, we investigated bias in conditionals and questions with a focus on the adverb *wirklich*, the modal verb *sollte*, and different sentence types (*ob* vs. *wenn*) and conditional subtypes (*falls* vs. *wenn/*V1) in German. We hypothesized that *sollte/wirklich/falls* all create negative bias about the antecedent proposition. The two experiments we conducted show effects in the predicted directions. One of the clearest bias generating effects we observed was the effect of the metaevaluation function *O* (Giannakidou & Mari, 2018a, b, 2021a, b) exhibited by *wirklich*. In addition, we also found differences between the *wenn*-conditionals and the *ob*-questions in Experiment 1 and between the *wenn-, falls*- and V1-conditionals in Experiment 2, with *wenn-* and V1-conditionals being similar in this aspect. Our studies are among the first that address bias in conditionals (in comparison to questions), and we expect our findings to deepen the understanding of what bias is, and how it is produced both generally and specifically in conditionals.

## Data Availability

All data and code associated with the experiment reported in this paper are available at the following data repository: https://osf.io/qkvtf/.

## Appendix 1

Below we list the target items [(item number), each item with S1/S2/S3/S4] in Condition 1 used in our experiments. Experiment 1 used item 1–40, presented in 8 conditions as in (23). Experiment 2 used items 1–45, presented in 9 conditions as in (26). Both experiments used the same list of 48 filler items in addition.

1. Susanne studiert Jura./Sie denkt: „Wenn ich Anwältin werde, mache ich eine eigene Anwaltskanzlei auf.“/Glaubt Susanne, dass sie Anwältin wird?/Studiert Susanne Mathe?
2. Paul macht gerade ein Marathon-Training./Er denkt: „Wenn ich die Strecke schaffe, laufe ich den nächsten Marathon mit.“/Glaubt Paul, dass er die Strecke schafft?/Macht Paul ein Krafttraining?
3. Nicole hat ein schwaches Immunsystem./Sie denkt: „Wenn Mario meine Jacke dabei hat, kann ich sie anziehen.“/Glaubt Nicole, dass Mario ihre Jacke dabei hat?/Hat Nicole ein starkes Immunsystem?
4. Samuel macht Urlaub am Meer./Er denkt: „Wenn ich am Montag frei bekomme, bleibe ich einen Tag länger.“/Glaubt Samuel, dass er am Montag frei bekommt?/Macht Samuel Urlaub in den Bergen?
5. Paula denkt über ihre Zukunft nach./Sie denkt: „Wenn ich eine Arbeit finde, kaufe ich mir ein Mac Book.“/Glaubt Paula, dass sie eine Arbeit findet?/Denkt Paola über Ihre Oma nach?
6. Marcel muss oft abends lange arbeiten./Er denkt: „Wenn ich Freitagabend frei habe, gehe ich mit meinen Freunden ins Kino.“/Glaubt Marcel, dass er Freitagabend frei hat?/Hat Marcel lange Arbeitszeiten?
7. Lena denkt über ihre Karriere nach./Sie denkt: „Wenn ich eine Beförderung bekomme, mache ich eine Feier.“/Glaubt Lena, dass sie eine Beförderung bekommt?/Denkt Lena über ihren Werdegang nach?
8. Carmen arbeitet gerne im Garten./Sie denkt: „Wenn ich ein Haus mit Garten bekomme, werde ich Rosen anpflanzen.“/Glaubt Carmen, dass sie ein Haus mit Garten bekommt?/Arbeitet Carmen gerne im Garten?
9. Christian spielt Fußball./Er denkt: „Wenn meine Mannschaft die Meisterschaft gewinnt, rasiere ich meine Haare ab.“/Glaubt Christian, dass seine Mannschaft die Meisterschaft gewinnt?/Spielt Christian Handball?
10. Stefan hat eine Lactoseintoleranz./Er denkt: „Wenn ich meine Tabletten vergesse, sollte ich lactosehaltiges Essen vermeiden.“/Glaubt Stefan, dass er seine Tabletten vergisst?/Hat Stefan eine Lactoseintoleranz?
11. Felix hat Marias Backform verloren./Er denkt: „Wenn ich die Form wiederfinde, werde ich sie Maria sofort zurückgeben.“/Glaubt Felix, dass er die Backform wiederfindet?/Hat Felix Marias Uhr verloren?
12. Tabea plant einen Ausflug./Sie denkt: „Wenn ich eine ganze Woche frei bekomme, fahre ich sofort los.“/Glaubt Tabea, dass sie eine ganze Woche frei bekommt?/Plant Tabea einen Ausflug?
13. Melanie sucht nach einem Sommerkleid./Sie denkt: „Wenn ich ein schönes finde, kaufe ich es sofort.“/Glaubt Melanie, dass sie ein schönes Kleid findet?/Möchte Melanie warme Stiefel kaufen?
14. Jan ist ein wenig erkältet./Er denkt: „Wenn morgen mein Training ausfällt, werde ich erleichtert sein.“/Glaubt Jan, dass sein Training morgen ausfällt?/Ist Jan erkältet?
15. Finn hat nächste Woche frei./Er denkt: „Wenn meine Schwester frei hat, gehen wir zusammen in die Bar.“/Glaubt Finn, dass seine Schwester frei hat?/Hat Finn nächste Woche frei?
16. Mark freut sich über seinen freien Tag./Er denkt: „Wenn die Bachelorette heute im Fehrnsehen läuft, bleibe ich zu Hause.“/Glaubt Mark, dass die Bachelorette heute im Fernsehen läuft?/Muss Mark heute zur Arbeit?
17. Tim verbringt das Wochenende zu Hause./Er denkt: „Wenn ich Besuch bekomme, werde ich einen Apfelkuchen backen.“/Glaubt Tim, dass er Besuch bekommt?/Verbringt Tim das Wochenende zu Hause?
18. Phillipp möchte etwas essen./Er denkt: „Wenn die letzte Vorlesung ausfällt, koche ich mein Lieblingsgericht.“/Glaubt Philipp, dass die letzte Vorlesung ausfällt?/Möchte Philipp etwas essen?
19. Sophia mag gerne ins Theater gehen./Sie denkt: „Wenn ich Karten für das neue Stück bekomme, lade ich meine Mutter ein.“/Glaubt Sophia, dass sie Karten für das neue Stück bekommt?/Geht Sophia gerne ins Theater?
20. Luisa möchte mit jemandem ins Kino gehen./Sie denkt: „Wenn meine Nachbarin zu Hause ist, kommt sie bestimmt mit.“/Glaubt Luisa, dass ihre Nachbarin zu Hause ist?/Möchte Luisa schwimmen gehen?
21. Albert geht gerne schwimmen./Er denkt: „Wenn das Schwimmbad geöffnet ist, gehe ich schwimmen.“/Glaubt Albert, dass das Schwimmbad geöffnet ist?/Geht Albert gerne schwimmen?
22. Mario möchte Socken stricken./Er denkt: „Wenn die Wolle ausreicht, stricke ich zwei Paare.“/Glaubt Mario, dass die Wolle für zwei Paare Socken ausreicht?/Möchte Mario Handschuhe stricken?
23. Karl braucht Hilfe beim Zimmerstreichen./Er denkt: „Wenn mein Bruder am Wochenende Zeit hat, hilft er mir bestimmt.“/Glaubt Karl, dass sein Bruder am Wochenende Zeit hat?/Möchte Karl sein Zimmer streichen?
24. Ronja hat Lust auf ein Eis./Sie denkt: „Wenn die Eisdiele geöffnet hat, gönne ich mir einen großen Eisbecher.“/Glaubt Ronja, dass die Eisdiele geöffnet hat?/Würde Ronja gerade gerne Kuchen essen?
25. Janice kocht Mittag für ihre Freunde./Sie denkt: „Wenn ich Kichererbsen da habe, koche ich Curry.“/Glaubt Janice, dass sie Kichererbsen da hat?/Isst Janice Frühstück?
26. Lupita geht zu einer Hochzeit./Sie denkt: „Wenn ich ein Kleid in meiner Größe finde, kaufe ich es.“/Glaubt Lupita, dass sie ein Kleid in ihrer Größe findet?/Heiratet Lupita?
27. Irina backt einen veganen Käsekuchen./Sie denkt: „Wenn ich Cashews finde, kann ich das schaffen.“/ Glaubt Irina, dass sie Cashews findet?/Backt Irina einen Käsekuchen?
28. Malcolm geht Pilze sammeln./Er denkt: „Wenn ich Pfifferlinge finde, koche ich eine Soße.“/ Glaubt Malcolm, dass er Pfifferlinge findet?/Geht Malcolm Pilze sammeln?
29. Pablo muss Geschenke kaufen./Er denkt: „Wenn ich einen Krimi finde, kaufe ich ihn für meinen Bruder.“ /Glaubt Pablo, dass er einen Krimi findet?/Muss Pablo Geschenke kaufen?
30. Amalia geht heute zur Schule./Sie denkt: „Wenn ich den Bus verpasse, fahre ich mit meiner Mutter mit.“/Glaubt Amalia, dass sie den Bus verpasst?/Hat Amalia heute frei?
31. Grace ist Opersängerin./Sie denkt: „Wenn Lisa heute Zeit hat, singe ich zwei Lieder.“/Glaubt Grace, dass Lisa heute Zeit hat?/Ist Grace Popsängerin?
32. Issa hat viele Prüfungen./Sie denkt: „Wenn ich alle Prüfungen bestehe, werde ich gut abschneiden.“/Glaubt Issa, dass sie alle Prüfungen besteht?/Muss Issa aufräumen?
33. Andre putzt seine Fenster./Er denkt: „Wenn das Putzmittel leer ist, fahre ich in die Stadt.“/Glaubt Andre, dass das Putzmittel leer ist?/Putzt Andre seine Fenster?
34. Jules trifft seinen alten Freund./Er denkt: „Wenn Nico Vegetarier ist, werde ich mich freuen.“/Glaubt Jules, dass Nico Vegetarier ist? /Trifft Jules seinen Freund?
35. Eleonora sucht ihr Ladekabel./Sie denkt: „Wenn ich es verloren habe, muss ich ein neues kaufen.“/Glaubt Eleonora, dass sie ihr Ladekabel verloren hat?/Sucht Eleonora ihr Ladekabel?
36. Jamila ist Schauspielerin./Sie denkt: „Wenn ich zum Casting eingeladen werde, bekomme ich die Rolle.“/Glaubt Jamila, dass sie zum Casting eingeladen wird?/Ist Jamila Lehrerin?
37. Lukas plant seine Geburtstagsfeier./Er denkt: „Wenn ich einen guten Termin finde, lade ich alle meine Freunde zu einer großen Party ein.“/Glaubt Lukas, dass er einen guten Termin findet?/ Plant Lukas seine Geburtstagsfeier?
38. Elisa ist in der Schule./Sie denkt: „Wenn der Feueralarm losgeht, muss ich schnell mit den Kindern aus dem Gebäude gehen.“/Glaubt Elisa, dass der Feueralarm losgeht?/Arbeitet Elisa im Kindergarten?
39. Marie möchte sich frei nehmen./Sie denkt: „Wenn es mit dem Urlaub klappt, werde ich einige Bücher lesen.“/Glaubt Marie, dass es mit dem Urlaub klappt?/Möchte Marie sich frei nehmen?
40. Gabriel geht mit seiner Frau einkaufen./Er denkt: „Wenn der Tofu im Angebot ist, kaufe ich mehrere Packungen.“/Glaubt Gabriel, dass der Tofu im Angebot ist?/Geht Gabriel allein einkaufen?
41. Lydia sucht ihre Handtasche./Sie denkt: „Wenn ich sie verloren habe, muss ich meine Frau anrufen.“/Glaubt Lydia, dass sie ihre Handtasche verloren hat?/Sucht Lydia ihre Jacke?
42. Mohammed plant seine Hochzeit./Er denkt: „Wenn der schöne Saal verfügbar ist, wird alles klappen.“/Glaubt Mohammed, dass der schöne Saal verfügbar ist?/Plant Mohammed seine Hochzeit?
43. Anton ordnet seine Dokumente./Er denkt: „Wenn ich heute damit fertig werde, trinke ich ein Glas Wein.“/Glaubt Anton, dass er heute damit fertig wird?/Ordnet Anton seine Dokumente?
44. Natasha geht ins Fitnessstudio./Sie denkt: „Wenn meine Kollegin mitkommt, probiere ich ein neues Gerät aus.“/Glaubt Natasha, dass ihre Kollegin mitkommt?/Geht Natasha ins Theater?
45. Sieba will einen Tanzkurs besuchen./Sie denkt: „Wenn es in der Gruppe freie Plätze gibt, melde ich mich an.“/Glaubt Sieba, dass es in der Gruppe freie Plätze gibt?/Will Sieba einen Kochkurs besuchen?

## Footnotes

1 There is also *contextual bias*, or bias relating to evidence available in the context (‘evidential bias’, Büring & Gunlogson, 2000; Romero & Han, 2004; Sudo, 2013; Northrup, 2014; Domaneschi et al., 2017), or ‘answer bias’ (Krifka, 2015; Malamud & Stephenson, 2015). We assume that such ‘biases’ are part of the belief basis of the speaker upon asking the question, i.e., the speaker is biased because contextual evidence allows it. We will not worry about how belief is formed (see Giannakidou & Mari, 2021a, 2021b for recent discussion on belief formation).

2 The Giannakidou and Mari notion of commitment we use is subjective, i.e., it represents the set of propositions held by *individual* linguistic agents (e.g., the speaker, the subject of the sentence in embedding which Giannakidou (1997) and Giannakidou & Mari call *individual anchors,* recycling a term from Farkas, 1992), it therefore differs from Krifka’s use of ‘commitment state’ which is “modelled as a set of propositions, containing the propositions that are *publicly shared by the participants* [emphasis ours]” (Krifka, 2015: 328–329). Krifka’s commitment corresponds to common ground assumptions, and the goal of speech acts is to “change a commitment state”. Speaker commitment in our view can be entirely private, even solipsistic (Giannakidou & Mari, 2016, 2018a, 2018b, 2021a, 2021b), as is the case with verbs of belief (but also imagination, and dreaming).

3 Native speakers we consulted have different intuitions on this.

4 Experimental evidence is provided for the *wenn/falls* contrast in Liu (2019, 2021). In addition, Liu (2019) also reports experimental evidence for the effect of NPIs related to negative bias in both English (the tested NPIs were *ever/at all*) and German (the test NPIs were *jemals/überhaupt*). The results for *überhaupt* were similar to those for *jemals*, which challenges the non-NPI based accounts, for example, the QUD-based account of Rojas-Esponda (2014). We leave the question of whether they are compatible for future work.

5 https://en.wikisource.org/wiki/The_Art_of_Literature/On_the_Study_of_Latin (accessed on 12. March 2021).

6 https://www.facebook.com/dasakademieberlin/posts/wenn-er-wirklich-im-lotto-gewinnen-sollte-fress-ich-einen-besen-dasakademie-lang/3147640535260523/ (accessed on 12. March 2021).

7 如果他要真的很喜欢你, …他就应该克服一下。 https://www.zhihu.com/question/316447372 (accessed on 14. June 2021).

8 It must be noted that native speakers diverge on whether *falls* can be used in counterfactual conditionals, and there may be some variation.

9 Original text: „Was man aber schon jetzt sagen kann, ist, dass *sollte* in etwa das Gleiche leistet wie *falls*: *falls* ist ebenfalls auf die Einleitung strikt hypothetischer Konditionale beschränkt und schließt kontrafaktischen Gebrauch aus; oft werden *falls*-Konditionale zusätzlich durch *sollte* markiert (Zifonun et al., 1997: 2281). Insbesondere verweist *falls* via seine wörtliche Bedeutung ,*für den Fall dass …* ‘ genau wie *sollte* explizit auf die Möglichkeit der Realisierung der Antecedens-Proposition.“

10 *Really* and its equivalents *wirklich* etc. are not morphologically modal adverbs in the sense that they are derived from a modal word. We will get back to the status of *wirklich* in Sect. 5.

11 Note that the bias can be bouletic (i.e., in terms of dispreferences), which we will not discuss in this paper.

12 We did not add random slopes because models including them failed to converge.

13 https://grammis.ids-mannheim.de/systematische-grammatik/2101 (accessed on 5. April 2021).

14 https://grammis.ids-mannheim.de/systematische-grammatik/2101 (accessed on 5. April 2021).

15 We did not add random slopes because models including them failed to converge.

16 https://context.reverso.net/übersetzung/deutsch-englisch/wir+das+Spiel+gewinnen (accessed 13. March 2021).

17 We thank a reviewer for pointing this out to us.
